# How Does the Public Perceive the Functions of Non‐Suicidal Self‐Injury? An Analysis by Gender and Age Group

**DOI:** 10.1002/npr2.70088

**Published:** 2026-01-18

**Authors:** Masaru Takahashi

**Affiliations:** ^1^ Faculty of Core Research Ochanomizu University Tokyo Japan

**Keywords:** non‐suicidal self‐injury, perceived function, public awareness, public perception, suicide prevention

## Abstract

Non‐suicidal self‐injury (NSSI) is a prevalent phenomenon among adolescents and poses significant public health concerns. Research has identified various functions of NSSI. However, public perceptions of these functions remain unclear. This cross‐sectional study explored public understanding of the functions of NSSI and examined the relations between these perceptions and demographic factors of gender and age group. A nationwide online survey was conducted with 2000 Japanese adults (mean age 44.6 years, SD = 14.3) to assess their agreement with 20 statements about NSSI functions using a six‐point scale. After conducting an exploratory factor analysis of the functions of NSSI, the study performed a two‐factor analysis of variance with the factor scores for each function as the dependent variable and gender and age group as the independent variables. The exploratory factor analysis revealed a four‐factor structure: (1) negative emotion regulation, (2) interpersonal effect, (3) avoidance of obligations, and (4) positive mood improvement. A series of two‐way analyses of variance revealed gender and age differences, with women and younger individuals being more likely to endorse emotion regulation functions, and middle‐aged and older adults more likely to support avoidance functions. Regarding interpersonal relationship factors, no statistically significant results were observed for either gender or age group. The findings suggest the importance of understanding public perceptions of NSSI functions for targeted psychological education and awareness. Future research should directly compare public perceptions with the perceptions of those who engaged in NSSI, considering other demographic factors.

## Abstract 2

Non‐suicidal self‐injury (NSSI) is a widespread public health concern, yet how the general public understands its functions has remained unclear. In a cross‐sectional online survey of 2000 Japanese adults, exploratory factor analysis of agreement with 20 NSSI‐function statements revealed four underlying dimensions—negative emotion regulation, interpersonal effect, avoidance of obligations, and positive mood improvement—and two‐way ANOVAs showed that women and younger individuals more strongly endorsed emotion regulation while middle‐aged and older adults favored obligation‐avoidance, with no demographic differences for interpersonal effects. These results underscore the importance of demographic‐sensitive psychoeducational initiatives and suggest future studies directly compare public perceptions with those of individuals who engage in NSSI.

## Graphical abstract

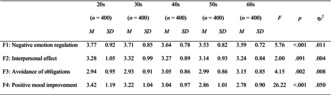

## 1 Introduction

Non‐suicidal self‐injury (NSSI) is a phenomenon that represents a significant public health concern. According to a recent meta‐analysis, the prevalence of NSSI among adolescents is relatively high, with approximately one in five to six adolescents affected [1, 2]. A history of NSSI has been shown to increase the risk of future suicide attempts [3, 4]. Consequently, clinicians must identify adolescents who repeatedly engage in NSSI at the earliest possible stage and facilitate their connection to mental health services for appropriate intervention.

Although much of the existing literature on NSSI originates from Western contexts, recent studies have underscored the importance of examining NSSI within culturally specific frameworks. In Japan, NSSI has increasingly been recognized as a pressing concern, particularly among adolescents. Prior research suggests that stigma surrounding mental health and self‐injurious behavior remains pervasive, and it is often shaped by cultural norms that discourage emotional disclosure and help‐seeking [5]. These sociocultural dynamics may influence both the prevalence and public understanding of NSSI, highlighting the need for context‐sensitive research and intervention strategies.

To understand individuals who engage in NSSI and to consider appropriate support and intervention strategies, clinicians must accurately ascertain the function and perceived benefits of the behavior. Extensive research has demonstrated that NSSI serves various functions [6, 7, 8, 9, 10]. These findings have provided valuable insights into the interpersonal and intrapersonal aspects of NSSI. Studies have shown that NSSI may serve interpersonal functions, such as eliciting attention and avoiding social situations. Additionally, research has established that NSSI fulfills crucial intrapersonal functions related to emotional regulation, including managing intense psychological distress, alleviating tension, and regulating overwhelming emotions. Notably, meta‐analytic findings indicate that the intrapersonal function of emotional regulation is more prevalent [11]. Accurately comprehending the function of NSSI is imperative in clinical practice. Such understanding informs the development of tailored treatment plans, facilitates targeted interventions, promotes empathy and understanding, enhances risk assessment and management, improves communication with stakeholders, and mitigates the escalation of harmful behaviors.

Researchers have also identified various misconceptions about NSSI that lack empirical support, many of which pertain to its function. For instance, a prevalent misconception is that NSSI is performed solely to elicit attention. This belief is widespread among professionals working in the human‐service field, including school teachers [12], medical staff [13, 14], and correctional facility staff [15], and is also associated with demographic factors. As previously delineated, NSSI serves multiple functions, with the most prominent ones aiming to alleviate intense emotional states such as severe anxiety, anger, and depression—rather than to seek attention. Furthermore, self‐injury is typically conducted in isolation and without disclosure to others, which challenges the notion that it is primarily attention‐seeking behavior. Stigma constitutes a significant impediment to the therapeutic objectives of clinical psychology [16]. Moreover, individuals who more strongly endorse the idea that NSSI serves interpersonal functions tend to exhibit more discriminatory responses [17].

While health care professionals' perceptions of NSSI have been examined in a growing body of research, studies investigating broader public perceptions of NSSI motivations and functions remain limited [18]. A possible discrepancy in perception between those who engage in NSSI and those around them may lead to inadequate support provision or detrimental misunderstandings. Research has demonstrated that societal prejudice toward NSSI is a significant deterrent for individuals seeking to disclose their experiences and pursue assistance [19]. In the Japanese context, such stigma may be further compounded by cultural norms that discourage emotional disclosure and help‐seeking. A large‐scale study of Japanese adolescents who self‐harmed found that approximately 40% did not seek help from anyone, despite experiencing psychological distress—often due to the absence of a perceived confidant and internalized beliefs that seeking help would burden others [5]. These findings underscore the importance of culturally sensitive approaches to NSSI education and intervention, particularly in collectivist societies where indirect communication and emotional restraint are normative. Enhancing public understanding of NSSI—especially within culturally specific frameworks—may foster more supportive environments for disclosure, reduce stigma, and promote timely engagement with formal support systems. Such efforts are essential for mitigating the persistence of NSSI and improving mental health outcomes across diverse populations.

A uniform awareness of NSSI functions among the public cannot be presumed. When considering psychological education and awareness‐raising initiatives for the public, stakeholders must examine the characteristics that predispose individuals to endorse certain beliefs about NSSI functions. Research on suicide‐related myths has demonstrated that being men and being over 60 years old are associated with a greater likelihood of endorsing misconceptions [20]. However, few studies have investigated the influence of gender and age group on estimates of NSSI function. Conducting such research could yield valuable insights for targeted psychoeducational interventions. Therefore, this study aimed to investigate how the public perceives the potential functions of NSSI and explore the relation between these perceptions and the demographic factors of gender and age group.

## 2 Methods

### 2.1 Participants

A total of 2000 adult residents of Japan participated in the study. The participants comprised 1000 men and 1000 women. They were recruited through an online research firm that requires panel members to submit government‐issued identification documents at the time of registration. While the firm verifies participants' place of residence, it does not collect information on nationality.

Given that the survey was administered in Japanese and recruitment was limited to residents of Japan, it is reasonable to assume that most participants were Japanese nationals. However, because nationality was not explicitly confirmed, the sample may include a small proportion of non‐Japanese residents.

Following an initial screening process to exclude individuals who did not demonstrate sufficient attentiveness in their responses (as detailed in the next section), recruitment continued until the target number of 200 participants per combination of age group (20s, 30s, 40s, 50s, and 60s) and gender (men, women) was reached, resulting in a total sample size of 2000 participants. The final sample had a mean age of 44.6 years (SD = 14.3), with ages ranging from 20 to 69 years.

### 2.2 Procedure

The data were collected in mid‐December 2023 using an online survey platform. Prior to participation, respondents were presented with a clear statement outlining the voluntary nature of the study, their right to withdraw at any time without penalty, and the assurance of anonymity. Submission of the completed questionnaire was considered as informed consent.

To ensure data quality, three attention‐check items were embedded at random points throughout the survey. These items instructed participants to select a specific response and were designed to identify inattentive respondents or those who may not have engaged with the survey content in a cognitively deliberate manner.

In anticipation of potential emotional discomfort, participants were informed in advance that certain items might address sensitive psychological topics. They were explicitly given the option to skip any question and to discontinue participation at any point. As an additional ethical safeguard, a list of mental health resources and helplines was provided at the conclusion of the survey for individuals who experienced distress or required support.

### 2.3 Measures

#### 2.3.1 Demographic Variables

The online panel had preregistered attribute information, which was used to ascertain the participant's gender and age group.

#### 2.3.2 Potential Functions of NSSI

To examine the level of public awareness regarding the potential functions of NSSI, participants were presented with 20 statements reflecting commonly reported motivations for engaging in NSSI, as identified in prior empirical studies [6, 7, 8, 9, 10]. Each statement was rated on a six‐point Likert scale ranging from 1 (strongly disagree) to 6 (strongly agree), providing a measure of perceived relevance and endorsement (see Appendix 1). The selected functions were adapted from prior studies on self‐reported reasons for NSSI and reflect a range of psychological and interpersonal motivations. Notably, Nock and Prinstein [10] proposed a widely cited functional model that organizes NSSI behaviors along two orthogonal dimensions: the type of reinforcement (positive vs. negative) and the locus of reinforcement (automatic/intrapersonal vs. social/interpersonal). This framework yields four distinct functional domains, encompassing behaviors aimed at reducing aversive internal states (automatic negative reinforcement), generating desired internal experiences (automatic positive reinforcement), escaping unwanted social situations or demands (social negative reinforcement), and obtaining interpersonal attention or support (social positive reinforcement). These domains provide a theoretically grounded basis for interpreting the diverse functions of NSSI and for assessing public awareness of its psychological complexity.

### 2.4 Statistical Analysis

Initially, the study computed the descriptive statistics for each item, including the mean and standard deviation for each gender and age group. Subsequently, an exploratory factor analysis was performed to aggregate the items. Finally, to investigate the influence of demographic variables on support for NSSI functions, the study conducted a series of two‐way analyses of variance between participants, using the scores for each factor as the dependent variable and gender and age group as the independent variables. All statistical analyses were conducted using version 29 of IBM SPSS Statistics for Windows.

## 3 Results

### 3.1 Exploratory Factor Analysis of NSSI Functions

The results showed a relatively low mean score for all items. The highest mean score was observed for both men and women regarding the statement “to express feelings that cannot be put into words” (*M* = 3.90, SD = 1.06). This was followed by “to ease anxiety” for women (*M* = 3.89, SD = 1.05) and “to make others aware of my suffering” for men (*M* = 3.64, SD = 1.12). The item with the lowest mean score overall was “to relax” (*M* = 2.85, SD = 1.17).

To elucidate the functions of self‐injurious behavior, the study conducted exploratory factor analysis (maximum likelihood method, Promax rotation) on the 20 items. Based on the attenuation of the eigenvalues and interpretability of the factors, the study determined a four‐factor structure as the most appropriate. Subsequently, after iterative factor analyses, eliminating an item (“to punish oneself”) with absolute factor loadings below 0.40 and those with high loadings on multiple factors, the study derived a four‐factor structure composed of 19 items.

Table 1 presents the factor pattern and inter‐factor correlations following Promax rotation. Factor 1 consisted of eight items, such as “To stop unpleasant feelings,” and was designated as the “negative emotion regulation” factor (*α* = 0.891). The second factor consisted of five items, such as “To attract the attention of others,” and was designated as the “interpersonal effect” factor (*α* = 0.890). The third factor included four items, such as “To avoid meeting others,” and was designated as the “avoidance of obligations” factor (*α* = 0.831). The fourth factor contained two items, such as “To relax,” and was designated as the “positive mood improvement” factor (*α* = 0.757). Based on the factor analysis results, the study then used the mean scores for the items that exhibited high loadings on each factor as the subscale scores. The alpha coefficients were calculated to assess the internal consistency of each scale. The obtained values ranged from 0.757 to 0.891 [21].

**TABLE 1:** Results for exploratory factor analysis of functions of non‐suicidal self‐injury.

	Items	Factor loading			
		1	2	3	4
*Factor 1: Negative emotion regulation*					
7	To express feelings that can't be put into words	0.83			
1	To stop unpleasant feelings	0.76			
5	To ease anxiety	0.73			
14	To forget worries	0.71			
15	Because physical pain is better than emotional pain	0.71			
9	To calm down when one is frustrated or angry	0.60			
10	To feel something, even if it's pain	0.59			
17	To stop feeling anything	0.50			
*Factor 2: Interpersonal effect*					
11	To attract the attention of others		0.88		
16	To get a reaction from people		0.86		
19	To surprise people around oneself		0.75		
13	To annoy those around oneself		0.71		
3	To make others aware of one's suffering		0.60		
*Factor 3: Avoidance of obligations*					
8	To avoid meeting others			0.76	
20	To avoid being scolded			0.72	
18	To avoid having to go to school or work			0.67	
4	To avoid doing things one doesn't want to do			0.63	
*Factor 4: Positive mood improvement*					
2	To relax				0.70
12	To feel refreshed				0.59
*Inter‐factor correlation*					
	F2	0.42	—		
	F3	0.47	0.60	—	
	F4	0.52	0.33	0.40	—

*Note:* Cronbach's α values are reported for each factor: Factor 1 = 0.891, Factor 2 = 0.890, Factor 3 = 0.831, Factor 4 = 0.757.

### 3.2 Two‐Way Analysis of Variance With the Dependent Variables of Four Functions

Tables 2 and 3 show the mean and standard deviation values of the four factor scores by gender and age groups, respectively. The study conducted a two‐way analysis of variance with each factor score as the dependent variable and gender and age group as the independent variables. The results indicated that the main effects of gender and age group were significant for the first factor (*F* (1, 1990) = 71.786, *p* < 0.001, *η*_*p*_^2^ = 0.035; *F* (4, 1990) = 5.764, *p* < 0.001, *η*_*p*_^2^ = 0.011), with women more likely to endorse negative emotion regulation function than men, and younger age groups demonstrating a greater tendency to support this function compared with middle and older age groups. For the third factor (avoidance of obligations), only the main effect of age group was observed (*F* (4, 1990) = 4.149, *p* = 0.002, *η*_*p*_^2^ = 0.008)—middle and older age groups demonstrated a greater tendency to support this function compared with younger age groups. The fourth factor (positive mood improvement) also showed the main effects of gender and age group (*F* (1, 1990) = 52.702, *p* < 0.001, *η*_*p*_^2^ = 0.026; *F* (4, 1990) = 26.224, *p* < 0.001, *η*_*p*_^2^ = 0.050)—women (more than men) and younger individuals (more than middle‐aged and older individuals) demonstrated a greater tendency to support this function. Regarding the second factor, the study found no significant differences for the main effects of gender and age group (*F* (1, 1990) = 1.938, *p* = 0.164, *η*_*p*_^2^ = 0.001; *F* (4, 1990) = 2.004, *p* = 0.091, *η*_*p*_^2^ = 0.004). Consistent with this, and across all four factors, no statistically significant interaction effects between gender and age group were observed.

**TABLE 2:** Descriptive statistics for four factor scores by gender.

	Men (*n* = 1000)		Women (*n* = 1000)		*F*	*p*	*η*_*p*_^2^
	*M*	SD	*M*	SD			
F1: Negative emotion regulation	3.49	0.81	3.80	0.81	71.79	< 0.001	0.035
F2: Interpersonal effect	3.22	0.95	3.28	0.93	1.94	0.164	0.001
F3: Avoidance of obligations	3.02	0.89	3.00	0.89	0.11	0.744	0.000
F4: Positive mood improvement	2.90	1.03	3.23	1.05	52.70	< 0.001	0.026

*Note:* *F, p*, and partial *η* ^2^ values are based on a two‐way between‐subjects ANOVA with gender and generation (five age groups) as fixed factors.

**TABLE 3:** Descriptive statistics for four factor scores by age group.

	20s (*n* = 400)		30s (*n* = 400)		40s (*n* = 400)		50s (*n* = 400)		60s (*n* = 400)		*F*	*p*	*η*_*p*_^2^
	M	SD	M	SD	M	SD	M	SD	M	SD			
F1: Negative emotion regulation	3.77	0.92	3.71	0.85	3.64	0.78	3.53	0.82	3.59	0.72	5.76	< 0.001	0.011
F2: Interpersonal effect	3.28	1.05	3.32	0.99	3.27	0.89	3.14	0.93	3.24	0.84	2.00	0.091	0.004
F3: Avoidance of obligations	2.94	0.95	2.93	0.91	3.05	0.86	2.99	0.86	3.15	0.85	4.15	0.002	0.008
F4: Positive mood improvement	3.42	1.19	3.22	1.04	3.04	0.97	2.86	1.01	2.78	0.90	26.22	< 0.001	0.050

*Note:* *F, p*, and partial *η* ^2^ values are based on a two‐way between‐subjects ANOVA with gender and generation (five age groups) as fixed factors.

## 4 Discussion

### 4.1 Higher Support for Emotional Regulation Among Women and Younger Individuals

Although the mean scores for all items were relatively low, the highest endorsement was observed for emotion‐related items such as “to express feelings that cannot be put into words.” This suggests that while the public may not broadly identify with most NSSI functions, emotional regulation is selectively recognized. Furthermore, the internal consistency of each factor was acceptable (*α* = 0.757–0.891), supporting the reliability of the derived structure for interpreting public perceptions [21].

The findings demonstrated that women were more likely to endorse the emotion regulation function of NSSI (i.e., “reducing negative emotions” and “increasing positive emotions”) compared with men. Additionally, younger individuals were more likely to support this function than middle‐aged and older adults. These results have several possible interpretations.

Research suggests that women typically exhibit a heightened awareness and expression of their emotions compared with men, particularly concerning negative emotions [22]. Consequently, women may be more receptive to the notion that NSSI functions as a means of emotional regulation. This perspective is associated with women's increased sensitivity to their emotions and the strategies they employ to manage them. Furthermore, societal expectations often encourage women to express their emotions more openly, which may account for women being more likely to cite emotional control as a reason for NSSI. Conversely, the same societal norms often expect men to suppress emotional vulnerability, making them less likely to acknowledge or recognize the emotional control function of NSSI. Overall, men and women may seek different functions from NSSI, which could, in turn, influence their estimation of these functions. Research indicates that compared with women, men exhibit significantly lower levels of emotional regulation function, while no gender differences in social functioning have been identified among individuals engaging in NSSI [23]. This finding is consistent with the results of the present study, which focused on the general population. The relatively high level of support for the emotional regulation function among young individuals may stem from their frequent experiences with intense emotional fluctuations, which increase the likelihood of perceiving NSSI as a mechanism for emotional control. In contrast to older individuals, young people are still in the process of developing their capacity to express and manage emotions. As individuals age, they develop alternative strategies for managing their emotions, which may account for the observed decrease in support for the emotional regulation function of NSSI with age.

### 4.2 Higher Support for Avoidance of Obligations Among Middle‐Aged and Older Adults

The findings suggest that middle‐aged and older adults are more inclined than younger individuals to view NSSI as a means to avoid obligations. This discrepancy may stem from generational differences in values, as older generations may have been raised in a societal context that prioritized fulfilling obligations over self‐expression. Consequently, they may interpret NSSI as a way to avoid responsibilities, overlooking its potential role in stress management or emotional regulation.

Moreover, such interpretations risk mistaking the context in which NSSI occurs for the actual reasons why individuals engage in it. Some adolescents may self‐injure in situations where they feel overwhelmed by obligations, such as school attendance or familial expectations. However, empirical research consistently indicates that these behaviors are primarily driven by internal emotional distress rather than strategic efforts to escape external demands. Thus, rather than serving as a calculated escape from obligations, NSSI typically functions as a way to manage intense and aversive emotional states. Klonsky and Glenn [24], in their psychometric validation of the Inventory of Statements About Self‐Injury, found intrapersonal functions such as affect regulation and self‐punishment to be more prevalent than interpersonal motives (e.g., obligation avoidance). Similarly, Nock and Prinstein's Four‐Function Model [10] demonstrated automatic negative reinforcement (e.g., reducing aversive internal states) as the dominant function, whereas social negative reinforcement (e.g., escaping interpersonal demands) was endorsed far less frequently. Supporting this view, Angelakis and Gooding's systematic review and meta‐analysis of 19 studies involving nearly 10 000 participants confirmed that NSSI was most strongly associated with attempts to alleviate internal suffering—such as anxiety, shame, or emotional numbness; conversely, social functions, including avoidance of obligations or attention‐seeking, showed significantly weaker associations [25].

Recently, mental health awareness has grown significantly [26], possibly leading to changes in how NSSI is understood—what was once stigmatized as recklessness or social avoidance is now recognized as a complex psychological response to distress. Research on NSSI indicates that social avoidance is not a predominant function compared with emotional regulation [8, 11, 25]. These findings, along with the results of this study, suggest a possible gap in perceptions of NSSI functions between individuals who engage in NSSI and older generations within the general population. Specifically, older generations may hold differing views or assumptions about the psychological and interpersonal functions that underlie NSSI behaviors, potentially leading to misunderstandings or stigmatization. Bridging this divide is essential for fostering empathetic, developmentally informed understandings of NSSI in both clinical and public discourse.

### 4.3 Practical Implications

The findings of this study underscore the heterogeneity of public perceptions regarding NSSI, which vary notably across gender and age. Prior research suggests that individuals lacking professional training often possess a limited understanding of the psychological underpinnings of NSSI, resulting in disproportionate reactions and diminished empathic responses [27]. Such perceptual discrepancies between individuals who engage in NSSI and those in their immediate social environments may hinder the provision of appropriate support and contribute to misinterpretations that exacerbate feelings of alienation and distress among affected individuals.

These observations point to an urgent need for public education initiatives that elucidate the complex and multifaceted nature of NSSI [28]. Specifically, the present findings highlight the value of targeted psychoeducational programs that are sensitive to demographic variables such as age and gender. Customizing these interventions to reflect the distinct perceptions and informational needs of diverse population segments may enhance their efficacy and social impact.

Translating these insights into practice requires structuring psychoeducational initiatives along the universal, selective, and indicated tiers of suicide prevention. Universal efforts—such as broad public campaigns and school‐based curricula—should be calibrated to age and gender to dispel misconceptions and underscore NSSI's primary emotion‐regulation function, thereby promoting early, non‐stigmatizing help‐seeking. Selective interventions should focus on groups prone to obligation‐avoidance beliefs (e.g., middle‐aged and older adults) through community workshops that reframe these interpretations as strategies for managing distress via guided dialogue and skills training. At the indicated level, clinical and crisis‐response materials must normalize help‐seeking, present alternative emotion‐regulation techniques, and engage family members to dismantle stigma and ensure timely support. By adopting demographic‐specific approaches—such as counselor‐led school programs for youth and professionally facilitated community sessions for older cohorts—and complementing them with compassion‐driven media campaigns, public health authorities can reshape public discourse, reduce stigma, and enhance pathways to care for those affected by NSSI.

### 4.4 Limitations

While this study makes a valuable contribution to the literature by quantifying public perceptions across a large sample in Japan, several limitations should be acknowledged. First, detailed demographic data—such as participants' place of residence, educational background, and household income—were not collected. Consequently, assessing the extent to which the sample reflects the broader Japanese population was not possible. Additionally, participants were recruited through an online panel, which may have excluded individuals with limited internet access or differing socioeconomic profiles. These sampling constraints may affect the generalizability of the findings, particularly with respect to regional, educational, and economic variation in attitudes and experiences. Future research should incorporate a more comprehensive set of demographic indicators and recruitment strategies to enable finer‐grained analyses of contextual diversity and sample representativeness.

Second, the study relied on self‐report measures, which are inherently vulnerable to response biases, such as social desirability bias or misinterpretation of survey items. Although efforts were made to mitigate these biases through attention checks, future research should consider incorporating alternative methodologies, such as qualitative interviews, to triangulate findings.

Third, the researcher examined the level of support for each function group among the public. By conducting a survey among individuals who frequently engage in NSSI and directly comparing the results, functions with existing gaps can be identified.

Fourth, the study was conducted exclusively in Japan, which limits its generalizability to other cultural contexts. Cultural norms and beliefs significantly influence perceptions of mental health and self‐injury; thus, the findings may not directly apply to other populations. Comparative studies involving diverse cultural backgrounds and societal structures would provide a more comprehensive understanding of how public perceptions of NSSI functions vary globally.

## 5 Conclusion

This study investigated how the Japanese public perceived the potential functions of NSSI and examined the relation between these perceptions and demographic factors of gender and age group. The study derived four factors to elucidate the functions of self‐injurious behavior, namely, the negative emotion regulation, interpersonal effect, avoidance of obligations, and positive mood improvement factors. The results indicated higher support for the emotional regulation function among women and younger individuals and higher support for the avoidance of obligations function among middle‐aged and older adults.

Based on these findings, the present study offers valuable insights into public perceptions of NSSI functions and underscores the importance of targeted educational efforts. By framing social applications within a three‐tiered prevention model—universal, selective, and indicated—demographic trends can be translated directly into practice. Universal campaigns can enhance mental‐health literacy across all groups; selective community programs can address obligation‐avoidance interpretations among older cohorts; and indicated materials can support high‐risk individuals and their families by presenting emotion‐regulation alternatives and reducing stigma. Finally, clearly targeting those who lack mental health literacy in information campaigns will help reduce the stigma attached to NSSI, which will in turn make it easier for those affected by NSSI to ask for help, leading to suicide prevention.

## Author Contributions

The author was responsible for all aspects of this research, including conceptualization, methodology, data collection, analysis, and manuscript preparation.

## Funding

This work was supported by the Innovative Research Program on Suicide Countermeasures of the Japan Suicide Countermeasures Promotion Center (Grant R4‐2‐2).

## Ethics Statement

This study was conducted according to the principles of the Declaration of Helsinki and was approved by the Humanities and Social Sciences Research Ethics Committee of the Ochanomizu University (No. 2023‐135).

## Consent

Submitting responses was considered as consent to participate.

## Conflicts of Interest

The author declares no conflicts of interest.

## Supporting information


**Data S1:** This file contains the response data used in the present study regarding the perceived functions of non‐suicidal self‐injury. The variable numbers for function correspond to the numbering of the items listed in the Appendix. All variables are coded using Likerttype scales.

## Data Availability

The raw data that support the findings of this study are available in the Supporting Information of this article.

## Appendix 1

The following is a list of survey questions used in this study. Each statement was designed to evaluate respondents' perspectives on the functions of non‐suicidal self‐injury.

1. Self‐injury is done “to stop unpleasant feelings”
2. Self‐injury is done “to relax”
3. Self‐injury is done “to make others aware of one's suffering”
4. Self‐injury is done “to avoid doing things one doesn't want to do”
5. Self‐injury is done “to ease anxiety”
6. Self‐injury is done “to punish oneself”
7. Self‐injury is done “to express feelings that can't be put into words”
8. Self‐injury is done “to avoid meeting others”
9. Self‐injury is done “to calm down when one is frustrated or angry”
10. Self‐injury is done “to feel something, even if it's pain”
11. Self‐injury is done “to attract the attention of others”
12. Self‐injury is done “to feel refreshed”
13. Self‐injury is done “to annoy those around oneself”
14. Self‐injury is done “to forget worries”
15. Self‐injury is done “because physical pain is better than emotional pain”
16. Self‐injury is done “to get a reaction from people”
17. Self‐injury is done “to stop feeling anything”
18. Self‐injury is done “to avoid having to go to school or work”
19. Self‐injury is done “to surprise people around oneself”
20. Self‐injury is done “to avoid being scolded”
